# Allostatic Load Measurement: A Systematic Review of Reviews, Database Inventory, and Considerations for Neighborhood Research

**DOI:** 10.3390/ijerph192417006

**Published:** 2022-12-18

**Authors:** Shawna Beese, Julie Postma, Janessa M. Graves

**Affiliations:** 1 College of Agricultural, Human, and Natural Resources Sciences, Washington State University, Pullman, WA 99164, USA; 2 College of Nursing, Washington State University, Spokane, WA 99202, USA

**Keywords:** allostasis, biomarkers, residence characteristics, review

## Abstract

Background: Neighborhoods are critical to understanding how environments influence health outcomes. Prolonged environmental stressors, such as a lack of green spaces and neighborhood socioeconomic disadvantage, have been associated with higher allostatic load levels. Since allostatic load levels experienced earlier in life have stronger associations with mortality risk, neighborhoods may be uniquely suited to monitor and mitigate the impacts of environmental stressors. Researchers often study allostatic load in neighborhoods by utilizing administrative boundaries within publicly accessible databases as proxies for neighborhoods. Methods: This systematic review of reviews aims to identify commonly used biomarkers in the measurement of allostatic load, compare measurement approaches, inventory databases to study allostatic load, and spotlight considerations referenced in the literature where allostatic load is studied in neighborhoods. The review was conducted using the search term “allostatic load” in the MEDLINE, CINAHL, and PsychINFO databases. The search results were filtered to include reviews. Results: The search returned 499 articles after deduplication. Overall, 18 synthesis reviews met the inclusion criteria and were retained for extraction. The synthesis reviews analyzed represented 238 studies published from 1995 to 2020. The original ten biomarkers were most often used to measure allostatic load. More recently, body mass index and C-reactive protein have additionally been frequently used to measure allostatic load burden. Conclusions: The scientific contributions of this study are that we have identified a clear gap in geographic considerations when studying allostatic load. The implication of this study is that we have highlighted geographic concepts when conducting neighborhood-level research using administrative databases as a neighborhood proxy and outlined emerging future trends that can enable future study of allostatic load in the neighborhood context.

## 1. Introduction

Neighborhoods are a setting of research interest for studies that seek to understand how environmental stressors influence future health outcomes. Neighborhoods are where we grow, live, work, and play [1,2]. Since the impacts of allostatic load on all-cause and cardiac-specific mortality are more strongly associated with allostatic load levels experienced earlier in life [3], neighborhoods may be uniquely suited to address the long-term health risks associated with high allostatic load levels. Perceived neighborhood quality [4,5] with increased neighborhood green spaces [6,7], decreased neighborhood disorder [8], and neighborhood socioeconomic advantage [9,10] have all been associated with normal allostatic load. Researchers often study allostatic load in the neighborhood context by utilizing administrative boundaries within publicly accessible databases as proxies for neighborhoods, which may not reflect the actual realities of the people who live in those neighborhoods [11,12].

Allostatic load conceptualizes the biosocial mechanisms of the prolonged activation of the acute stress response [13]. Allostatic load has been conceptually well-established in scientific literature as reflecting the overall wear-and-tear on the body from environmental stresses, and it is considered a precursor of chronic disease development [14,15,16,17]. An initial battery of 10 biomarkers was reported in the seminal work to quantify allostatic load and has since been utilized in research as a cumulative indicator of overall stress adaptation [15,16]. The initial allostatic load battery is composed of four primary mediators (representing biochemical changes in the neuroendocrine system as the stress response is initiated) and six secondary mediators (representing structural remodeling of receptors sites of the cardiovascular, immune, and metabolic systems due to long-term activation of the stress response). The original primary mediators included cortisol, noradrenaline (norepinephrine), adrenaline (epinephrine), and dehydroepiandrosterone (DHEA). These mediators are responsible for triggering the hypothalamic–pituitary–adrenal axis and the sympathetic–adrenal medullary axis cascades of the stress response or are an outcome of the primary cascades. Secondary mediators measure the symptom manifestations of a prolonged stress response. The original six secondary mediators include systolic blood pressure (SBP), diastolic blood pressure (DBP), waist-to-hip ratio (WHR), high-density lipoprotein (HDL), total cholesterol (TC), and glycosylated hemoglobin (HgbA1C) [15,16].

Throughout the initial decades of allostatic load being used in research to conceptualize overall stress burden, the use of biomarkers intended to represent allostatic load became much more varied from study to study [18,19]. In addition, the concept started to be explored as a measure of episodic trauma and not cumulative wear-and-tear over the life-course [20]. Additionally, the utility of allostatic load in settings outside of the initial setting of longevity studies also became prominent [6,21,22,23].

Social epidemiologists started to explore allostatic load as a potential conceptualization of how place-based determinants of health impact the life course of health [24]. Similarly, spatial epidemiologists wanted to better understand how allostatic load interplays with the neighborhood context to determine health. Although the focus on the allostatic load in the neighborhood context is increasing in the literature and doctoral dissertations, a more standardized approach to measuring allostatic load, especially when studying neighborhoods, is needed to compare studies and appraise the overall inferences in the body of literature [25,26]. 

### Aims

This systematic review conceptualizes the allostatic load as a precursor to chronic disease development. This study aims to determine which biomarkers are frequently used in research to measure allostatic load and to provide researchers with database tools to examine allostatic load and the geographic considerations for studying allostatic load in the neighborhood context. To address these aims, this systematic review focuses on evaluating systematic reviews in published, peer-reviewed health sciences literature.

## 2. Materials and Methods

The design of this study is a systematic review of reviews (also termed an “umbrella review”). The search strategy for this approach focuses on the identification and examination of systematic reviews, meta-analyses, and other forms of synthesized literature. A systematic review of reviews serves as an accepted and valuable method to gain a clear understanding of the specific aspects of a broad topic area [27].

The inclusion criteria for this study were that each included article was a meta-analysis, systematic review, or high-quality synthesis review. Other inclusion criteria included the following: English language, publication in a peer-reviewed journal, human subjects, and use of biomarkers to measure allostatic load. Animal studies were excluded.

Since the first two of the three aims were focused on the measurement of allostatic load as a general concept, we did not limit the search terms by geography. Using the search term “allostatic load,” a search was conducted on 6 July 2021, in the following databases: MEDLINE (through PubMed), CINAHL (through EBSCOhost), and PsychINFO (through the American Psychological Association). The results were filtered by the inclusion criteria described above and limited to reviews published between 2003 and 2021. We knew from previous searches that the first synthesis review was published in 2003 and the search was conducted in 2021. The search results were imported into Covidence [28], a software program for systematic reviews, and independently screened by investigators for the inclusion criteria. Two investigators (S.B., J.M.G.) independently reviewed the articles to assess if the inclusion criteria were met. The articles included in the study are hereafter referred to as synthesis reviews. 

The following information was extracted from each synthesis review: the review type, the number of studies reviewed, the purpose of the study, the population addressed, and whether potential conflicts of interest were addressed. Additional information extracted from each synthesis article included biomarkers used to determine the biomarker frequency, the measurement approach for allostatic load, and the databases used in the study. Each database identified in the synthesis reviews was searched to determine whether neighborhood measures were available in each database. This systematic review was not registered. All the study procedures adhered to the PRISMA 2020 Statement Checklist.

### Quality Appraisal

Protocols to eliminate selection bias are essential in systematic reviews seeking to inform practice [29,30]. The AMSTAR-2 was used as the quality appraisal tool in this study [30]. This tool, designed to aid in the critical appraisal of systematic reviews that synthesize healthcare studies, is validated with reviews that include randomized control and non-randomized control trials that evaluate health, such as observational studies [31].

Cohen’s Kappa was utilized to determine interrater reliability. Covidence™ software (Veritas Health Innovation Ltd., Melbourne, Australia) was utilized to calculate Cohen’s Kappa. We reconciled any discrepancies in the quality appraisals of the AMSTAR-2 scoring through consensus. To determine the overall quality of the synthesis reviews, we calculated the total scores using the AMSTAR-2 tool. The responses “Yes,” “Partial,” and “No,” were assigned numeric values, so “Yes” = 1, “Partial” = 0.5, and “No” = 0 (maximum possible score: 16). Synthesis reviews with AMSTAR-2 scores within the interquartile range were considered “average” quality, while those with scores below and above the interquartile range were classified as “weak” and “strong,” respectively. 

## 3. Results

**Figure 1:**
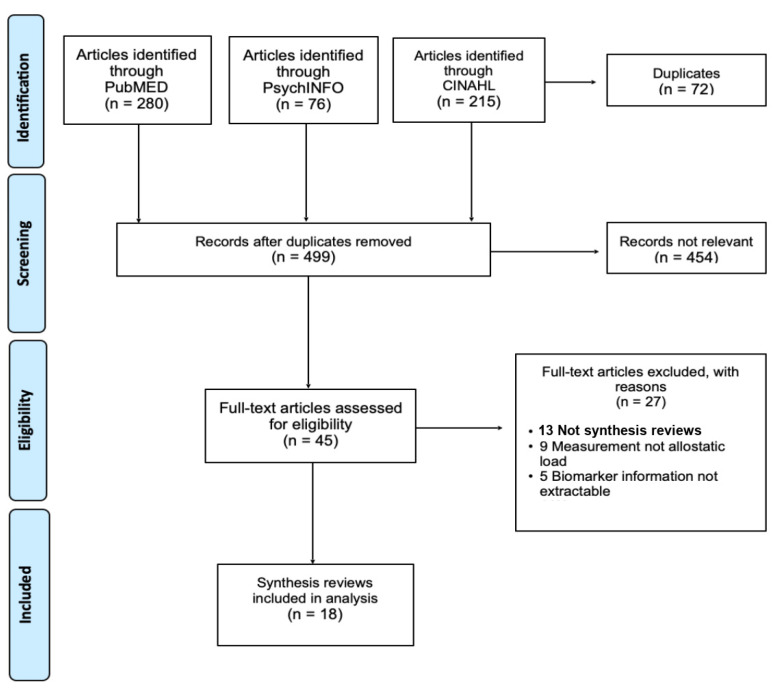
PRISMA Algorithm for the systematic literature search for “allostatic load” conducted on 6 July 2021.

The initial search returned 571 articles with 72 duplications (Figure 1). Two investigators independently screened the title and abstract of 499 articles and full-text reviews of 45 articles. Overall, 18 synthesis reviews met the inclusion and exclusion criteria and were extracted for analysis. These synthesis reviews represent 238 studies published from 1995 to 2020. All 238 individual studies were cross-referenced across the synthesis review to remove duplicates. There were cases where individual studies were included in multiple synthesis reviews; however, the biomarkers of the 238 individual studies were only accounted for once in the analysis.

### 3.1. Interrater Reliability

Cohen’s Kappa was used to determine the agreement between the two independent reviewers for the relevance screening of the articles using the title/abstract and full-text review to assess whether the inclusion criteria were met. There was moderate agreement between the reviewers for the title/abstract screening phase (Cohen’s κ = 0.533) and almost perfect agreement for the full-text reviews (κ = 0.82). We resolved discrepancies in the rating through discussion and consensus. 

### 3.2. Characteristics of the Included Reviews

Ten out of eighteen synthesis reviews self-identified as systematic reviews (*n* = 8) or systematic reviews combined with meta-analyses (*n* = 2). Other article types included as synthesis reviews were literature reviews (*n* = 3), perspective reviews (*n* = 3), a scoping review (*n* = 1), and a narrative review (*n* = 1). We analyzed the individual studies described in each synthesis review (range: 6 to 61 studies, mean: 19, median: 23.3). Potential conflicts of interest were explicitly addressed in 66.6% of the synthesis reviews by explicitly stating no conflicts of interest exist or including a statement of the conflicts of interest with an explanation. In total, 33.3% of the synthesis reviews did not acknowledge a conflict of interest in any way within the text of the manuscript. The purpose and description of the population of each synthesis review are summarized in Table 1. Only one of the synthesis reviews included any geographic considerations for studying allostatic load.

### 3.3. Quality of Research and Potential Bias

We used AMSTAR-2 to individually appraise each of the 18 synthesis reviews (Table 1). The AMSTAR-2 scores of the synthesis reviews ranged from 1.5 to 12.5, with a mean of 5.9 (standard deviation: 3.0, interquartile range: 9.0–3.0). Two-thirds (66.6%) of the synthesis reviews were classified as average (scores within the interquartile range), while 22.2% and 11.1% were classified as weak and strong, respectively. The synthesis reviews classified as weak failed to account for bias in the inclusion and exclusion protocols, did not include how bias was addressed, or explicitly stated potential conflicts of interest. The reviews rated as strong explicitly used pre-stated protocols for the inclusion criteria, provided a robust description of the role of bias in selecting individual studies, and included a quality appraisal of the individual studies reviewed.

To mitigate potential bias for this umbrella review, we developed a formal protocol with defined inclusion and exclusion criteria prior to conducting our search. We used two independent reviewers for a title/abstract screen and full-text review. All conflicts of interest were disclosed. 

### 3.4. Biomarker Frequency

**Figure 2:**
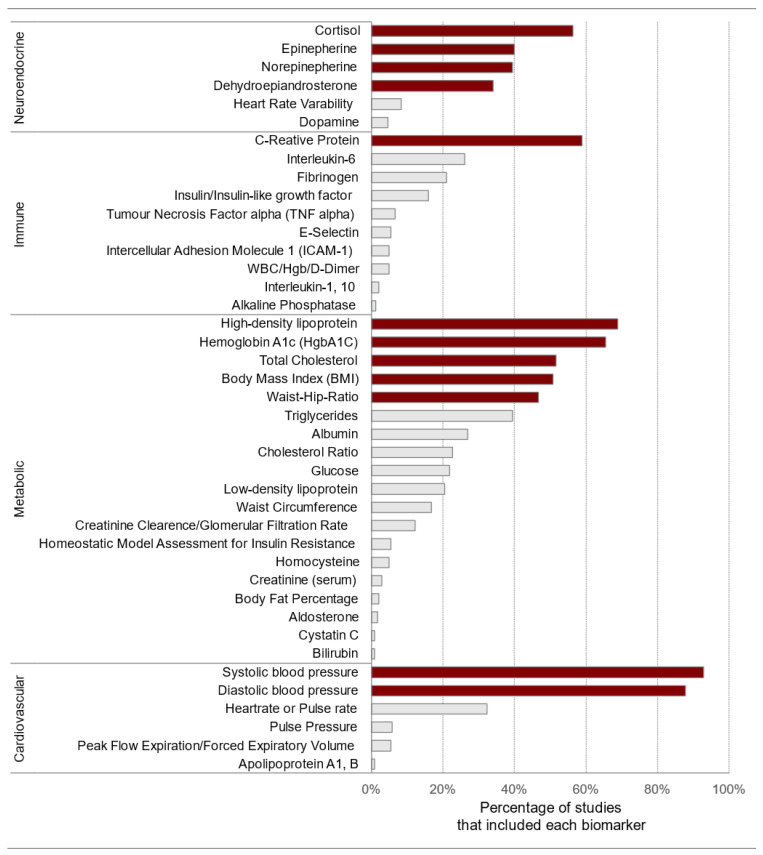
Biomarker use frequency extracted from 18 synthesis reviews identified through a systematic literature search for “allostatic load” conducted on 6 July 2021. The darker shaded biomarkers are the original 10, body mass index, and C-reactive protein. Note: HgbA1C = glycosylated hemoglobin; white blood cell count (WBC), hemoglobin (Hgb).

The most frequently utilized primary mediator was cortisol (*n* = 134). Cortisol was used in 56.3% of the 238 studies represented in the 18 synthesis reviews (Figure 2). Epinephrine was used in 39.9% (*n* = 95) and norepinephrine in 39.4% (*n* = 94) of the individual studies represented in our analysis. Dehydroepiandrosterone was used in 34.0% (*n* = 81) of the extracted studies. The final two primary mediators that were used in relatively few studies over the years, but were not part of the original biomarkers, were heart-rate variability (*n* = 20, 8.4%) and dopamine (*n* = 11, 4.6%).

The most frequently used secondary mediators included cardiovascular biomarkers of systolic blood pressure (*n* = 221, 92.8%) and diastolic blood pressure (*n* = 209, 87.8%). The lesser-used cardiovascular biomarkers that appeared in the literature were heart rate/pulse rate (*n* = 77, 32.3%), pulse pressure (*n* = 14, 5.8%), peak flow expiratory (*n* = 13, 5.4%), and apolipoprotein A1, B (*n* = 2, 0.8%). Among the metabolic system biomarkers, the most frequently used were high-density lipoprotein (*n* = 164, 68.9%), glycosylated hemoglobin (*n* = 156, 66.6%), total cholesterol (*n* = 123, 51.6%), body mass index (*n* = 121, 50.8%), and waist-to-hip ratio (*n* = 111, 46.6%). C-reactive protein (*n* = 140, 58.8%), interleukin-6 (*n* = 62, 26.0%), and fibrinogen (*n* = 50, 21.0%) were the most frequently used immune system biomarkers. In addition to these metabolic and immune biomarkers, there were 14 more metabolic biomarkers with a frequency of usage ranging from 39.4% to 0.8% and 7 additional immune biomarkers with a frequency of usage ranging from 15.9% to 1.2%.

Traditionally, two primary approaches have been used to calculate allostatic load scores. A frequently used method to calculate allostatic load is to evenly weigh all the biomarkers in a composite measure representing the sum of the measured biomarkers, whereby each is coded as “0” for normal/low findings or “1” for high findings (per nationally established ranges). High allostatic loads are determined by summing the score. High allostatic load risk is determined when three or more biomarkers are in high-risk ranges [11,18]. The most frequently used method of calculation is some variation of the original calculation of using extreme quantiles, such as the 10/90th percentile, per biomarker to determine the acceptable range. There is no consistent quantile cut-off; the researchers of individual studies describe the percentiles for all the biomarkers and justify the cut-off defined by the research team [18,19,32,33]. A third method used is to use z-scores; however, this calculation method is used far less frequently than the two primary methods. A wide variation persists among the studies included in the synthesis reviews if calculation methods were examined and discussed.

**Table 1:** Characteristics of the analyzed articles extracted from 18 synthesis article reviews identified through a systematic literature search for “allostatic load” conducted on 6 July 2021.

**References**	Review Type	Number of Studies	AMSTAR2 Quality	Purpose of Review	Population Addressed	Conflicts of Interest Addressed
Szanton et al., 2005 [34]	Systematic review	10 [15,35,36,37,38,39,40,41,42,43]	Average	Examine and synthesize the literature on allostatic load as a construct to understand and qualify health disparities.	Target population is unspecified.	Yes
Dowd et al., 2009 [44]	Systematic review	7 [35,36,42,45,46,47,48]	Average	Review of the existing literature on socioeconomic status and cortisol, as well as allostatic load.	Target population is unspecified.	Yes
Juster et al., 2010 [49]	Literature review	59 [15,16,35,36,37,39,40,41,42,43,45,46,47,48,50,51,52,53,54,55,56,57,58,59,60,61,62,63,64,65,66,67,68,69,70,71,72,73,74,75,76,77,78,79,80,81,82,83,84,85,86,87,88,89]	Average	Review of the existing literature on the theoretical and empirical work that exists on allostatic load.	Target population is unspecified.	No
Beckie, 2012 [90]	Systematic review	58 [15,16,35,36,37,38,39,40,41,42,43,45,46,48,49,53,58,59,60,61,62,63,64,67,68,69,74,75,77,78,82,83,84,85,86,87,88,91,92,93,94,95,96,97,98,99,100,101,102,103,104,105,106,107,108,109,110]	Average	Review of the existing literature to synthesize the state of the science on allostatic load over the life span.	Target population variable and specified by study.	Yes
Mauss et al., 2015 [111]	Systematic review	16 [40,67,73,74,75,78,82,84,104,112,113,114,115,116]	Average	Examine and synthesize literature on allostatic load measurement, operational measures, and biomarkers used in measurement.	Target population is workers, context specified by studies.	No
Duong et al., 2017 [18]	Perspective review	20 [48,54,58,80,87,93,95,100,117,118,119,120,121,122,123,124,125,126,127,128]	Weak	Review of the calculation of allostatic load studies that used NHANES data 1988 and 2010.	Target population variable and specified by study.	Yes
Johnson et al., 2017 [19]	Literature review	26 [35,36,73,81,87,89,94,101,113,114,120,122,128,129,130,131,132,133,134,135,136,137,138,139,140,141]	Average	Review of the biomarkers and methods used to analyze the association between allostatic load and socioeconomic position.	Target population is unspecified.	No
Rosemberg et al., 2017 [142]	Perspective review	12 [40,66,67,76,78,84,96,108,143,144,145]	Weak	Elucidate and synthesize the historical development of the allostatic load and its use in nursing research.	Two stress-vulnerable populations, workers, and women of childbearing age.	No
Wiley et al., 2017 [32]	Systematic review	24 [36,38,40,41,50,53,72,76,103,106,113,133,141,146,147,148,149,150,151,152,153,154,155,156]	Average	Explore and synthesize the literature on allostatic load and with psychological/social resources.	Target population is unspecified.	Yes
Ribeiro et al., 2018 [11]	Scoping review	14 [9,87,94,130,145,147,157,158,159,160,161,162,163,164]	Average	Explore and synthesize the literature on allostatic load and neighborhood socioeconomic deprivation.	Target population is unspecified.	Yes
Larrabee Sonderland et al., 2019 [165]	Systematic review	20 [38,41,47,53,72,76,103,133,141,147,152,153,155,156,166,167,168,169,170,171]	Average	Review of the relationship between everyday stress, social connectedness, and allostatic load.	Target population variable and specified by study.	Yes
D’Amico et al., 2020 [33]	Systematic review and meta-analysis	18 [15,16,37,59,63,98,118,172,173,174,175,176,177,178,179,180,181,182]	Strong	Investigate the association between allostatic load and standardized cognitive test among adults.	The target population is 18 and older.	Yes
Ketheesan et al., 2020 [183]	Narrative review	6 [184,185,186,187,188,189]	Weak	Explore and synthesize the literature on allostatic load and mental health disparities observed between indigenous and non-indigenous Australians.	Target population is indigenous Australians, age unspecified.	No
Kerr et al., 2020 [190]	Systematic review	61 [38,40,54,57,60,66,67,73,78,82,86,92,100,102,103,106,112,113,117,135,137,146,148,149,191,192,193,194,195,196,197,198,199,200,201,202,203,204,205,206,207,208,209,210,211,212,213,214,215,216,217,218,219,220,221,222,223,224,225,226,227]	Average	To assess sex differences in allostatic load and identify allostatic load associations that are specific to women.	Target population is women, ages specified by study.	Yes
Misiak, 2020 [228]	Perspective review	9 [202,215,229,230,231,232,233,234,235]	Weak	Explore and synthesize the literature on allostatic load and psychotic disorders.	People diagnosed with a psychotic disorder; characteristics specified by study.	No
Suvarna et al., 2020 [236]	Systematic review	24 [9,46,83,92,101,117,128,140,161,214,237,238,239,240,241,242,243,244,245,246,247,248,249,250]	Average	Examine and synthesize the literature on allostatic load and health-related behaviors.	Target population variable and specified by study.	Yes
Mathew et al., 2021 [251]	Systematic review and meta-analysis	12 [109,121,124,211,252,253,254,255,256,257,258,259]	Strong	Explore and synthesize the literature on allostatic load and cancer.	People diagnosed with cancer; characteristics specified by study.	Yes
Whelan et al., 2021 [260]	Literature review	24 [81,120,122,136,146,147,149,151,152,161,187,197,261,262,263,264,265,266,267,268,269,270,271,272]	Average	Identify and synthesize the literature on variations of allostatic load measurement and cancer.	Target population is adolescents.	Yes

### 3.5. Database Inventory

Multiple studies that were reviewed used secondary databases to determine allostatic load. We extracted those databases from the individual studies included in the synthesis reviews and inventoried them. A full description of each database, including the web address and the allostatic biomarkers the researcher can access in the database, is presented in Supplemental Material Table S1. The United States databases identified in the review were the Multi-Ethnic Study of Atherosclerosis (MESA) [273,274,275], the Community Child Health Research Network (CCHN) [276,277], Midlife in the United States (MIDUS) [278], the Jackson Heart Study [279], and the National Archive of Computerized Data on Aging (NACDA) [280]. The databases that provide georeferenced biomarker data include the National Health and Nutrition Examination Survey (NHANES) [281], and the Health and Retirement Study (HRS) [282].

## 4. Discussion

This study aimed to identify the frequency of biomarkers used to measure allostatic load in the published literature. Unsurprisingly, the findings demonstrated that the original ten biomarkers were most often used to measure allostatic load. More recently, body mass index and C-reactive protein biomarkers have started being used to reflect metabolic pathways and inflammation.

A detailed account of how allostatic load was calculated by each of the individual studies reviewed within the synthesis review was provided in 13 of the 18 synthesis reviews included in this study. One comment, “The 21 studies calculated ALS (allostatic load score) in 18 different ways using 26 different biomarkers” fairly characterized the findings from all the synthesis reviews that examined allostatic load calculations. There is wide variation in how allostatic load is calculated; however, we have summarized the most frequently used methods found for each of the synthesis reviews (noted in Supplemental Materials Table S1).

### 4.1. Geographic Consideration in Neighborhood Research

A secondary aim of this systematic review was to spotlight geographic considerations for researchers conducting research at the neighborhood level. There was a marked gap in the literature regarding geographic considerations when studying allostatic load. Only one synthesis review, by Ribeiro et al., addressed geographic considerations from all the synthesis reviews included in this study. 

There are four common biases to be aware of when using administrative databases to study neighborhoods, two of which are noted in the literature reviewed [11]. Administrative proxies include census data and areal-level public and private databases. First, ecological fallacies arise when inferences about the characteristics of one scale form the conclusions at a different scale (e.g., inferences about an individual based on neighborhood characteristics). Three criteria must be satisfied to establish an ecological fallacy: (a) the findings must result from the use of population data, (b) the results must be inferred to the individual level, and (c) there must be individual data that contradict the findings [283].

Second, the observations attributed to the neighborhood defined by administrative boundaries may differ from the actual neighborhood attributes. This difference is called the Modifiable Areal Unit Problem (MAUP). The MAUP is especially significant when using choropleths and should be addressed in the literature [284].

Third, a bias that can be created is the Uncertain Geographic Context Problem (UGCoP). This is the acknowledgement that using geographic (neighborhood) exposure context is not fully understood by the researcher. The amount of time individuals have lived in the studied neighborhood, the ground realities, and the amount of time individuals are exposed to environments that are not their neighborhood all need to be considered when interpreting neighborhood findings [11]. 

One last consideration for neighborhood researchers to be aware of was not found in our review but is nonetheless an important concept for neighborhood researchers. Tobler’s first law of geography states, “everything is related to everything else, but near things are more related than distant things” [285]. Spatial autocorrelation/dependence refers to the degree of autocorrelation that must be accounted for statistically. Assessing and adjusting for spatial dependence requires understanding the nature of clustering and dispersion patterns [286].

Several barriers to accessing georeferenced biomarker data exist. For example, restricted data-center application processes may require a time horizon of multiple months (this is the case for NHANES). The fees for accessing restricted data may also limit researchers’ ability to conduct geographic analyses using these data sources. For example, accessing NHANES via the Restricted Data Center requires a minimum application cost of USD 3000.

Additionally, the All of Us database [287], a racially, ethnically, and regionally diverse national database, is relatively new (initial release in 2018). This may explain why it was not identified in our analysis. Nonetheless, we included All of Us in our inventory as an additional tool for allostatic load researchers (noted in Supplemental Materials Table S2).

### 4.2. Future Trends in Allostatic Load Measurement

There is a movement within the allostatic load measurement community to operationalize allostatic load measurement for clinical application [288]. “Clinimetrics” represents the science of clinically applicable metrics [289]. Allostatic load clinimetrics can hold utility in both the primary care setting and the neighborhood context [290]. An example of a potential clinimetrics measurement would be the development of a clinical tool that explores the associations between Adverse Childhood Experience (ACE) scores and the development of high allostatic load [22,291]. Studies that examine both clinimetrics criteria and biomarkers present an exciting new realm of allostatic load inquiry.

Another horizon in allostatic load research is studying whether even weighting of biomarkers is the best method of allostatic load calculation. Are some biomarkers more predictive of overall wear-and-tear than others? A recent study that used the item response theory explored biomarker weighting using 2015–2016 NHANES data. The study concluded that body mass index and C-reactive protein were the most informative biomarkers [292].

The last horizon in allostatic load research is validity testing, which aims to use streamlined composites using fewer biomarkers. One recent study established that 5 biomarkers are highly associated with the allostatic load composite of the original 10 biomarkers [293]. The five streamlined biomarkers are diastolic blood pressure, glycosylated hemoglobin, low-density lipoprotein, waist circumference, and the heart-rate variability measure of the root mean square of the successive difference between normal heartbeats. This could be valuable for future research, as heart-rate variability is a biomarker that can be obtained from wearable devices. This reduces the laboratory testing to glycosylated hemoglobin and low-density lipoprotein.

### 4.3. Limitations

The primary limitation of this systematic review of reviews was choosing the AMSTAR-2 as our quality-appraisal tool. The AMSTAR-2 is designed to appraise systematic reviews of healthcare interventions. Many of the studies included in the synthesis reviews were observational studies and did include an intervention. However, we used the AMSTAR-2 because it fit most of the study designs and is a reputable/rigorous tool for appraising the quality of review literature.

## 5. Conclusions

This systematic review of reviews summarizes the current state of the art of measuring allostatic load. We identify the most frequently used biomarkers and present standardized practices for calculating allostatic load.

We include the historical perspective of allostatic load biomarkers and calculation variations and highlight crucial conceptual neighborhood considerations for studying allostatic load in neighborhood contexts. The inventory of public databases that include commonly used biomarkers is intended to facilitate future research on allostatic load. 

## Data Availability

Not applicable.

## Supplementary Materials

The following supporting information can be downloaded at https://www.mdpi.com/article/10.3390/ijerph192417006/s1, Table S1: Allostatic Load Calculation Methods extracted from 18 synthesis reviews identified through a systematic literature search for “allostatic load” conducted on 6 July 2021; Table S2: Database inventory extracted from 18 synthesis reviews identified through a systematic literature search for “allostatic load” conducted on 6 July 2021.
