# Supplementary material for: Allostatic Load Measurement: A Systematic Review of Reviews, Database Inventory, and Considerations for Neighborhood Research
Source: Int J Environ Res Public Health. 2022 Dec 18;19(24):17006. doi: 10.3390/ijerph192417006 (PMC9779615; doi:10.3390/ijerph192417006)
Supplement: Supplementary file 1 [file ijerph-19-17006-s001.zip › ijerph-2018077-supplementary.pdf]

Review

Allostatic Load Measurement: A Systematic Review of Reviews, Database Inventory, and Considerations for Neighborhood Research

Shawna Beese, Julie Postma and Janessa M. Graves

Supplemental Materials Table S1. Allostatic Load Calculation Methods extracted from 18 synthesis reviews identified through a systematic literature search for "allostatic load" conducted on 6 July 2021.

Reference	Was Allostatic Load Calculation Method Addressed?	Summary of Allostatic Load Calculation Methods Used
**Szanton et al., 2005**[34]	Yes	Most common method, allostatic load dichotomized biomarkers based on quartiles and then summed
**Dowd et al., 2009**[44]	No	Not addressed
**Juster et al., 2010**[49]	Yes	Most common method, allostatic load dichotomized biomarkers based on quartiles and then summed
**Beckie, 2012**[90]	Yes	Allostatic load dichotomized biomarkers based on clinical ranges or quartiles
**Mauss et al., 2015**[111]	Yes	A discussion regarding the strengths and limitations of the varied methods of allostatic load calculations
**Duong et al., 2017**[18]	Yes	Variations include a) sum of biomarker z-scores, b) sum of dichotomized biomarkers based on quartiles, and c) sum of dichotomized biomarkers based on clinical cutoffs
**Johnson et al., 2017**[19]	Yes	Allostatic load dichotomized biomarkers based on quartiles
**Rosemberg et al., 2017**[142]	Yes	Most common method, allostatic load dichotomized biomarkers based on quartiles and then summed
**Wiley et al., 2017**[32]	Yes	Most common method, allostatic load dichotomized biomarkers based on quartiles and then summed
**Ribeiro et al., 2018**[11]	Yes	Variations include a) sum of biomarker z-scores, b) sum of dichotomized biomarkers based on quartiles, and c) sum of dichotomized biomarkers based on clinical cutoffs

**Larrabee Sonderland et al., 2019**[165]	No	Not addressed
**D'Amico et al., 2020**[33]	Yes	Variations include a) sum of biomarker z-scores, b) sum of dichotomized biomarkers based on quartiles, and c) sum of dichotomized biomarkers based on clinical cutoffs
**Ketheesan et al., 2020**[183]	Yes	Allostatic load dichotomized biomarkers based on clinical ranges or quartiles
**Kerr et al., 2020**[190]	No	Not addressed
**Misiak, 2020**[228]	No	Not addressed
**Suvarna et al., 2020**[236]	No	Not addressed
**Mathew et al., 2021**[251]	Yes	Variations include a) sum of biomarker z-scores, b) sum of dichotomized biomarkers based on quartiles, and c) sum of dichotomized biomarkers based on clinical cutoffs
**Whelan et al., 2021**[260]	Yes	Biomarker summary scores with higher values indicating greater physiological strain.

Supplemental Materials Table S2. Database inventory extracted from 18 synthesis reviews identified through a systematic literature search for "allostatic load" conducted on 6 July 2021.

Database Name	Website	Biomarkers Available
	**Databases with Georeferenced Biomarker Data**	
National Health and Nutrition Examination Survey (NHANES)	https://www.cdc.gov/nchs/nhanes/index.htm	A1C, albumin, BMI, CRP, creatinine, DBP, fasting glucose, HR, PR, SBP, TC, TG, and WHR
Health and Retirement Study (HRS)	https://hrs.isr.umich.edu/about	A1C, CRP, Cystatin-C, DBP, HDL, HR, SBP, TC, and WC
	**Public Databases**	
All of Us Research Hub (discovered outside of the review)	https://www.researchallofus.org/	A1C, albumin, BMI, cortisol, CRP, creatinine, DBP, DHEA, Epinephrine (n <1000), HDL, Heart Rate, LDL, SBP, TC, and TG
Multi-Ethnic Study of Atherosclerosis (MESA)	https://www.mesa-nhlbi.org/	Glucose, HR, HWR, LDL, PP, SBP, and TG

Community Child Health Research Network (CCHN)	https://dash.nichd.nih.gov/study/1649	A1C, BMI, cortisol, CRP, DBP, HDL, LDL, SBP, and WHR
**Other Databases Cited in Review**		
MIDUS: Midlife in the United States	https://www.icpsr.umich.edu/web/pages/NACDA/midus.html	A1C, DBP, TC, cortisol, CRP, DHEA, epinephrine, e-selectin, fibrinogen, HRV, ICAM, IL-6, norepinephrine, and SBP
Jackson Heart Study	https://www.jacksonheartstudy.org/	A1C, albumin, BMI, CRP, creatinine, DBP, E-selectin, fasting glucose, HDL, HOMA-IR, WHR, HR, LDL, TC, and TG
National Archive of Computerized Data on Aging (NACDA)	https://www.icpsr.umich.edu/web/pages/NACDA/	Albumin, creatinine clearance, CRP, DBP, HDL, IL-6, SBP, TC, TC: HDL, and WHR

Note: A1C = glycosylated hemoglobin, BMI = body mass index, CRP = C-reactive protein, DBP = diastolic blood pressure, DHEA = dehydroepiandrosterone, HDL = high-density lipoprotein, HOMA-IR = homeostatic model assessment for insulin resistance, HR=heart rate, HRV= heart rate variability, ICAM=intracellular adhesion molecule, IL-6= interleukin 6, LDL= low-density lipoprotein, PP= pulse pressure, SBP=systolic blood pressure, TC= total cholesterol, TC:HDL= high-density lipoprotein to total cholesterol density, TG= triglycerides, WHR = waist to hip ratio, and WC= waist circumference. The darker shaded biomarkers are the original 10, BMI, and CRP.
